# Self-reported impulsivity does not predict response caution^☆^

**DOI:** 10.1016/j.paid.2020.110257

**Published:** 2020-12-01

**Authors:** Craig Hedge, Georgina Powell, Aline Bompas, Petroc Sumner

**Affiliations:** a CUBRIC - School of Psychology, Cardiff University, UK; b School of Psychology, Cardiff University, UK

**Keywords:** Response control, Inhibition, Impulsivity, Response caution, Diffusion model, UPPS-P, Self-control, Boundary separation

## Abstract

The broad construct of impulsivity is one that spans both personality and cognitive ability. Despite a common overarching construct, previous research has found no relationship between self-report measures of impulsivity and people's ability to inhibit pre-potent responses. Here, we use evidence accumulation models of choice reaction time tasks to extract a measure of “response caution” (boundary separation) and examine whether this correlates with self-reported impulsivity as measured by the UPPS-P questionnaire. Response caution reflects whether an individual makes decisions based on more (favouring accuracy) or less (favouring speed) evidence. We reasoned that this strategic dimension of behaviour is conceptually closer to the tendencies that self-report impulsivity measures probe than what is traditional measured by inhibition tasks. In a meta-analysis of five datasets (total *N* = 296), encompassing 19 correlations per subscale, we observe no evidence that response caution correlates with self-reported impulsivity. Average correlations between response caution and UPPS-P subscales ranged from rho = −0.02 to −0.04. While the construct of response caution has demonstrated value in understanding individual differences in cognition, brain functioning and aging; the factors underlying what has been called “impulsive information processing” appear to be distinct from the concept of impulsivity derived from self-report.

## 1 Introduction

The constructs of impulsivity and self-control play a prominent role in our current understanding of personality and neuropsychological disorders (Chambers, Garavan, & Bellgrove, 2009; Eysenck & Eysenck, 1985; Whiteside & Lynam, 2001). However, research into the definition and measurement of impulsivity continues to raise questions about whether the wide range of tasks and measures that are used do in fact measure some common underlying construct. Though different taxonomies of impulsivity exist, one fundamental distinction is that performance in behavioural tasks often shows little to no relationship with self-report measures (Creswell, Wright, Flory, Skrzynski, & Manuck, 2019; Cyders and Coskunpinar, 2011, Cyders and Coskunpinar, 2012; Eisenberg et al., 2019; Sharma, Markon, & Clark, 2014). Here, we try to bridge the gap using a cognitive model to isolate a dimension of cognition characterised as “impulsive information processing” (Metin et al., 2013), “decision urgency” or “response caution” (Evans, Rae, Bushmakin, Rubin, & Brown, 2017; Hedge, Powell, Bompas, Vivian-Griffiths, & Sumner, 2018).

One proposed reason for the discrepancy between questionnaires and behavioural tasks is that they were developed with different goals in mind. Cyders and Coskunpinar (2011) suggest that self-report measures focus on general tendencies or traits while lab-based tasks focus on “snapshots” of behaviour, which may be more sensitive to fluctuations in states (see also Wennerhold & Friese, 2020). For example, the UPPS-P impulsivity questionnaire consists of five subscales labelled negative urgency, positive urgency, (lack of) premeditation, (lack of) perseverance and sensation-seeking (Lynam, Smith, Whiteside, & Cyders, 2006). Individuals rate the extent of their agreement with statements about their general behaviour, such as “I am a cautious person”, an item in the premeditation subscale.

In contrast, behavioural impulsivity tasks are sometimes broadly categorised as either *impulsive choice* or *impulsive action* (Weafer & de Wit, 2014). Impulsive choice tasks typically consist of delayed gratification or gambling tasks, where individuals decide between uncertain or delayed large rewards and certain or immediate lower value rewards. Impulsive action typically refers to tasks designed to measure an individual's ability to rapidly inhibit a response to a salient or pre-potent stimulus (repeatedly). For example, in the Stroop task (Stroop, 1935) participants must quickly and accurately classify the font colour of a word while ignoring its meaning. Performance is traditionally measured by subtracting reaction times or error rates in a congruent or baseline condition (the word ‘red’ in red font) from an incongruent or conflict condition (the word ‘red’ in blue font). The subsequent RT cost or error cost is taken as an index of an individual's ability to overcome conflicting information. Further subcategories of inhibition tasks have also been proposed (Friedman & Miyake, 2004; Sharma et al., 2014), though these are not consistently supported by data (Rey-Mermet, Gade, & Oberauer, 2018). Note that the subtraction of performance in a baseline condition is often done for the explicit purposes of removing individual differences in confounding factors such as strategy or caution (Donders, 1969; though recent reviews have highlighted problems with this assumptions; Draheim, Mashburn, Martin, & Engle, 2019; Hedge, Powell, Bompas, et al., 2018).

**Fig. 1:**
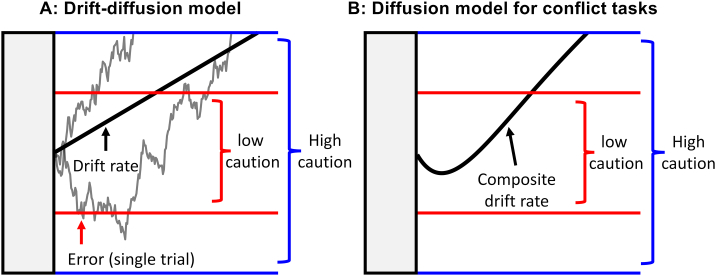
Schematic of two evidence accumulation models. A. In the drift diffusion model (Ratcliff, 1978), the decision on each trial (jagged lines) is represented by the noisy accumulation of evidence to a boundary. The solid black line represents the average rate of evidence accumulation or ‘drift rate’. The upper and lower boundary represent the correct and incorrect response respectively. An individual who sets a low boundary (red lines) waits for less evidence before responding and is more likely to make an error due to noise in the accumulation process. B: In the diffusion model for conflict tasks (Ulrich et al., 2015), the average rate of evidence accumulation is a composite of both controlled processing and automatic activation. The automatic activation function captures the assumption that prepotent response features (e.g., incongruent flankers) are processed via a fast, automatic route (Ridderinkhof, 2002). The solid black line shows the underlying accumulation for an incongruent trial, where automatic activation elicited from (e.g.) flankers in the flanker task contributes to the incorrect response tendency in the early part of the decision phase. See Supplementary material A for more information. (For interpretation of the references to colour in this figure legend, the reader is referred to the web version of this article.)

This conceptual distinction between the ability to overcome conflict and the general tendencies for cautious or impulsive behaviours may explain why low correlations are observed between inhibition tasks and self-report measures. We therefore reasoned that correlations might be uncovered if we could extract a measure of ‘caution’ from cognitive tasks. In our recent work, we have used evidence accumulation models to better understand individual differences in choice reaction time tasks, and conflict tasks in particular (Hedge, Powell, Bompas, et al., 2018; Hedge, Powell, & Sumner, 2018a; Hedge, Vivian-Griffiths, Powell, Bompas, & Sumner, 2019). The evidence accumulation framework represents a broad family of models that assume that the decision process can be represented by a process of sampling evidence from the stimulus until a boundary or threshold has been reached (see Fig. 1; for reviews see Donkin, Brown, Heathcote, & Wagenmakers, 2011; Forstmann, Ratcliff, & Wagenmakers, 2016; Ratcliff & Smith, 2004). These models can be used to dissociate and quantify the multiple processes that contribute to behaviour.

For our current purposes, we are particularly interested in the boundary separation parameter, sometimes referred to as “response caution”. In the evidence accumulation model framework response caution represents how much evidence an individual requires before they make a response. An individual with a preference for speed is assumed to set a lower boundary, such that they respond quickly but risk making more errors due to noise in the decision processes. In some studies, such individuals have been referred to as having “impulsive information processing” (Metin et al., 2013). In contrast, a cautious individual waits for more information before responding to ensure accuracy at the expense of reaction time, corresponding to a higher boundary.

The individual's preference for speed vs. accuracy is mathematically independent of the individual's *ability* to extract information and prime the desired response (while inhibiting or controlling unwanted information or response tendencies). This ability is represented by the rate that correct evidence accumulates towards the boundary, rather than the height of the boundary. In the drift-diffusion model (Ratcliff, 1978; Fig. 1A), the underlying rate of evidence accumulation (the drift rate) is assumed to be linear, though subject to moment-to-moment noise. Extensions of the diffusion model, such as the diffusion model for conflict tasks (Ulrich, Schroter, Leuthold, & Birngruber, 2015; Fig. 1B), assume that the information extracted from a stimulus can vary over time as a function of both automatic and controlled processes. This captures the assumption that prepotent response features (e.g., incongruent arrows in a flanker task) initially capture attention but that their influence decreases or is inhibited over time. But in either case, response caution (i.e., the boundary) is a separate parameter from these processes.

The quantitative dissociation of response caution from information processing efficiency (drift rate) has led to valuable insights into cognitive and behavioural changes in fields such as aging (Ratcliff et al., 2006, Ratcliff et al., 2010), as well as neuropsychological conditions such as autistic spectrum disorder and attention-deficit hyperactivity disorder (Karalunas et al., 2018; Pirrone, Dickinson, Gomez, Stafford, & Milne, 2017; Pirrone, Johnson, Stafford, & Milne, 2020; Powell et al., 2019). In most cases (though not all), older adults and individuals with autism show higher levels of response caution relative to young adults and healthy controls respectively. Interestingly, separate studies have shown higher levels of risk aversion in older adults when using self-report measures (the Domain-Specific Risk-Taking scale; Rolison, Hanoch, Wood, & Liu, 2014) and behavioural gambling tasks (Rutledge et al., 2016). Similarly, there is evidence that individuals with autism adopt more risk-averse strategies in gambling tasks (Gosling & Moutier, 2018; South et al., 2014). This co-occurrence of increased response caution in choice reaction time tasks and increased risk aversion in other domains hints at a possible underlying link related to the concept of impulsivity.

We have routinely included the UPPS-P questionnaire in our recent experiments, following completion of the behavioural tasks, with the overarching goal of examining whether using a cognitive model to dissociate caution from other cognitive mechanisms can reveal a relationship with impulsivity that was previously hidden. Here, we present a meta-analysis of the evidence we have accumulated over these studies (for a discussion of this approach, see Goh, Hall, & Rosenthal, 2016). We focus on response caution for the theoretical reasons above, and also because we have recently observed that this parameter correlated strongly across conflict tasks (average rho = 0.54), whereas the parameters associated with conflict and inhibition did not (rho = 0.04; Hedge, Powell, Bompas, & Sumner, 2020). We reasoned that if we do not see correlations with a parameter that is both theoretically aligned to impulsivity and that correlates well across tasks, then we are unlikely to see correlations with other sub-facets of cognition (Wennerhold & Friese, 2020). We examine all of the UPPS-P subscales in our analysis, though there is arguably most conceptual overlap between response caution and the items in the *lack of premeditation* and *perseverance* subscales, which refer to planning and attentiveness. Both these dimensions and *negative urgency* have also previously shown weak (*r* = 0.1) but significant correlations with traditional measures of response inhibition (Cyders & Coskunpinar, 2011), though these studies did not dissociate response caution from ability as we do here.

## 2 Method

### 2.1 Datasets

We conducted a new analysis of published data. These studies and their basic details are given in Table 1. We have previously reported the behavioural results and model parameters to examine whether the tasks themselves correlate with each other, but we have not previously reported the correlations between cognitive response caution (or the other model parameters) and the UPPS-P subscales that are the focus of our analysis here.

**Table 1:** Details of datasets included in meta-analysis. See Source publications for detailed information. The number of correlations is dependent on the number of boundary separation parameters estimated from the dataset (at least one per task). See Fig. 2 for a schematic of the tasks.

Dataset	Source	N	Tasks	Conditions	Trials per condition	Number of correlations
1	Hedge, Powell, et al. (2020)	50	Flanker	3	336	1
			Simon	3	336	1
2	Hedge et al. (2018b)	103	Flanker	3	480	1
			Stroop	3	480	1
3	Hedge, Powell, Bompas, et al. (2018)	102	Simon (blocked trials)	2	288	2^a^
			Simon (intermixed trials)	2	288	1
4	Hedge et al. (2019; Exp. 1)	43	Flanker	9	576	3^b^
			Stroop	9	576	3^b^
5	Hedge et al. (2019; Exp. 2)	69	Flanker	9	192	3^b^
			Dot motion	6	240	3^b^

Note. The data were collapsed across two separate testing sessions in Datasets 2 (three weeks apart) and 4 (four weeks apart).

a The blocked version of the task includes a separate boundary estimate for congruent and incongruent trials.

b Datasets include separate boundary estimates for blocks in which instructions emphasise either speed, accuracy, or both speed and accuracy.

We adopt a meta-analytic approach to maximise the power of these data to detect a meaningful correlation between caution and the UPPS-P subscales, should one exist. To assess this, we conducted a sensitivity power analysis for a random effects meta-analysis under varying assumptions of heterogeneity in the effect sizes (Griffin, 2020; Pigott, 2012). Based on 19 effect sizes and an average sample size of 68, we have 80% power to detect correlations equal to or greater than rho = 0.09, 0.11, and 0.16 assuming small, moderate, and large levels of heterogeneity respectively (α = 0.05, two-tailed).

### 2.2 Behavioural tasks

**Fig. 2:**
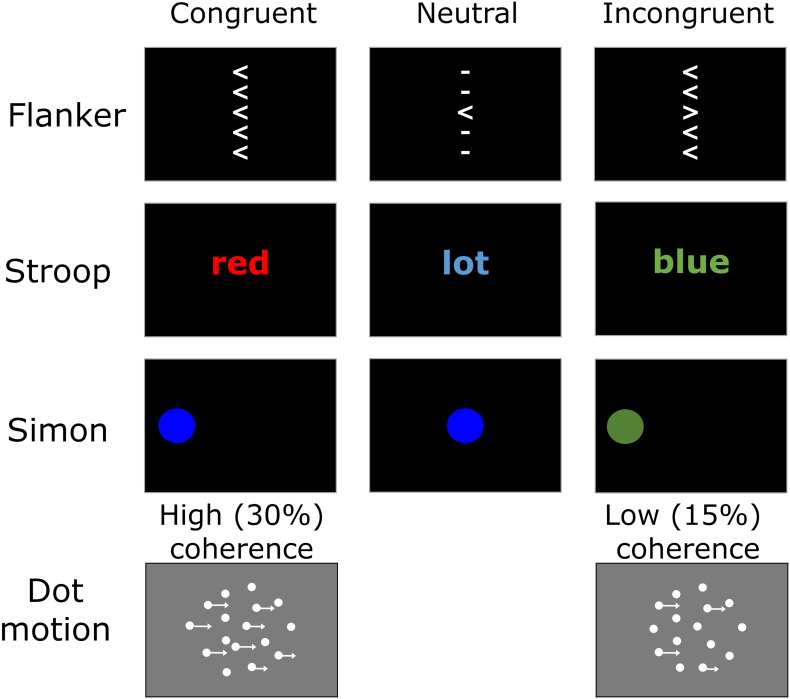
Schematic of the choice reaction time tasks used in all datasets. In the flanker task participants identify the central arrow as pointing to either the left or the right. In the Stroop task participants identified the font colour as red, blue, green or yellow. In the Simon task participants identified the circle as either blue or green. In the dot-motion task, participants identified whether the direction of coherent motion in an array of dots was to the left or right. Trials were separated by an interstimulus interval of 750 ms except in the dot motion task where the interstimulus interval was 500 ms. (For interpretation of the references to colour in this figure legend, the reader is referred to the web version of this article.)

Fig. 2 shows a schematic of the choice reaction time tasks used. In all tasks, participants were required to decide which of two (four in the Stroop) alternatives to categorise a stimulus. In the flanker task participants must respond to the direction of the central arrow and ignore the flanking stimuli. In the Stroop task participants must respond to the colour of the font and ignore the written word. In the Simon task participants must respond to the colour of the circle and ignore its location. In the dot motion task participants must respond to the direction of coherent motion in an array of dots. The Simon task in Dataset 3 did not include a neutral condition. All the tasks are commonly used to measure response inhibition except the dot motion task in Dataset 5, which is commonly used in both human and animal studies of decision making (c.f. Ratcliff & McKoon, 2008). Datasets 4 and 5 included a speed-accuracy trade-off manipulation, in which participants were instructed to prioritise speed, accuracy or both equally in separate blocks. In all other datasets, participants were instructed to be both as fast and as accurate as possible.

For our current purposes, the critical similarity between these tasks is that they all allow us to measure the level of caution with which individuals approach speeded decision making. We have previously observed moderate to strong between-task correlations in the response caution parameter (Hedge et al., 2019; Hedge, Powell, et al., 2020). Therefore, we interpret response caution to be a (at least in part) general process or set of processes. Here, where we are interested in the relationship between response caution and impulsivity, we treat the correlations from each task as an estimate of this same relationship.

### 2.3 UPPS-P impulsive behaviour scale

The UPPS-P is a 59-item questionnaire that measures five components of impulsivity: negative urgency, premeditation, perseverance, sensation seeking, and positive urgency (Lynam et al., 2006; Whiteside & Lynam, 2001). Participants rate their agreement with each item on a four-point Likert-type scale, ranging from Agree strongly (1) to Disagree Strongly (4). The subscales have shown high internal consistencies (>0.81; Whiteside & Lynam, 2001), and good to excellent three-week test-retest reliabilities for the subscales in this population (ICCs from 0.70 to 0.91; Hedge, Powell, & Sumner, 2018b). In our test-retest reliability studies (Datasets 2 and 4), participants completed the UPPS-P in both sessions. We average the subscale values across sessions for our correlations here.

### 2.4 Model fitting

For the conflict tasks in Datasets 1–5, we fit the diffusion model for conflict tasks (Ulrich et al., 2015) using Matlab. For the dot motion task in Dataset 5, we fit the standard drift-diffusion model using the DMAT toolbox in Matlab (Vandekerckhove & Tuerlinckx, 2008). We describe both models and their parameters in detail in Supplementary material A. Both models produce a boundary separation parameter which we interpret as response caution (see Fig. 1). The “number of correlations” column in Table 1 corresponds to the number of boundary separation parameters estimated from each task. For tasks with no instruction manipulation and where congruent, neutral and incongruent trials are randomly intermixed within blocks (Datasets 1 and 2), we obtained one boundary parameter per task. In the “blocked trials” variant of the Simon task in Dataset 3, we obtained separate estimates for congruent and incongruent trials. For the speed-accuracy trade-off experiments in Datasets 4 and 5, we obtained separate estimates for blocks where speed, accuracy, and both speed and accuracy were emphasised.

The details of our model fitting can be seen in Hedge, Powell, et al. (2020) for Datasets 1 to 4, and Hedge et al. (2019) for Dataset 5. Briefly, parameters are estimated by comparing reaction times for correct and error responses to data simulated from the model. We use optimisation algorithms to find the set of parameters that minimises the discrepancy between the observed and simulated data. Each participant and task are fit independently. We collapsed Datasets 2 and 4 across testing sessions for the purposes of model fitting. For the diffusion model for conflict tasks we adopted a common approach (c.f. White, Servant, & Logan, 2017) wherein we created six bins based on quantiles of the observed reaction times ([0.1, 0.3, 0.5, 0.7, 0.9]), and counted the number of trials in each bin. We did this separately for correct and error responses, and separately for each condition. When participants made fewer than eleven errors in that condition, error reaction times were instead grouped into three bins ([0.3, 0.5, 0.7]). When fewer than five errors were made, we fit the median reaction time. We initially simulated data from the model using 5000 sets of randomly generated parameters and compared the deviance (−2 log-likelihood) between the observed data and each of our simulated datasets. We then submitted the best 15 parameter sets to a Nelder-Mead simplex (Nelder & Mead, 1965) based algorithm in Matlab, which we restarted 3 times to avoid local minima. The correlations we entered into our meta-analysis are with the best fitting parameters for each participant and task derived from this process. A similar approach is implemented in DMAT (Vandekerckhove & Tuerlinckx, 2008), which we used to fit the standard drift diffusion model to the dot-motion task (Dataset 5).

We examined the four-week test-retest reliability for the conflict diffusion model for the speed-accuracy trade-off tasks in Dataset 4 (Hedge et al., 2019). For boundary separation, these ranged from poor (ICC = 0.39) to good (ICC = 0.71). These values fall within the range observed elsewhere in the literature with the standard drift-diffusion model across a variety of tasks (Enkavi et al., 2019; Lerche & Voss, 2017; Schubert, Frischkorn, Hagemann, & Voss, 2016). To our knowledge, there is currently no evidence as to whether there are systematic differences in the reliability of parameters derived from different tasks or administration methods. For our current purposes, these findings suggest that there is some stability in individuals' levels of response caution.

### 2.5 Meta-analysis of caution with impulsivity

For each dataset, we calculated Spearman's rho correlations between each boundary separation estimate and each UPPS-P subscale. For example, in Dataset 1 we computed correlations between the UPPS-P and both boundary separation in the flanker task and boundary separation in the Simon task. This produced 19 correlations in total across the datasets. These correlations were then meta-analysed using a multilevel random effects meta-analysis, implemented in the metafor package in R (R Core Development Team, 2017; Viechtbauer, 2010). We fit a three-level random effects model, which allows us to account for non-independence of correlations taken from the same dataset. At the first level, we assume that there is variance in the effect size estimates due to sampling error. At the second level, we assume that there is variance in the effect sizes that we are trying to estimate within each dataset. As we take multiple correlations from the same dataset, we refer to these as being nested within datasets. At the third level, we assume variance across datasets. To assess heterogeneity, we examined the I^2^ statistic, which represents the percentage of total variance attributable to between- and within-dataset variance relative to sampling variance (Viechtbauer, 2019).

We report the individual correlations, and the results for other parameters, in Supplementary material B. The data and analysis code are available from the Open Science Framework (https://osf.io/w8va9/).

## 3 Results

**Fig. 3:**
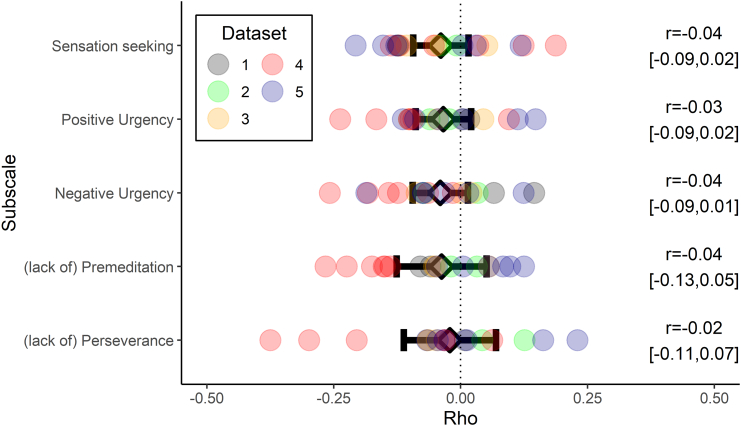
Meta-analytic (black diamonds) and observed (circles) correlations between boundary separation/response caution and the UPPS-P impulsivity questionnaire subscales. Error bars and brackets show 95% confidence intervals. A multi-level random effects meta-analysis was performed on Spearman's rho correlations calculated for each pair of tasks, allowing for clustering where multiple correlations were taken from the same dataset. Note that all the 95% confidence intervals include zero.

High scores on the UPPS-P subscales are indicative of higher impulsivity, whereas lower values of boundary separation are associated with lower caution. Therefore, if there were a consistent link between impulsivity and caution, we would expect correlations to be consistently negative. Fig. 3 plots the main results from our meta-analysis. For every UPPS-P subscale, the 95% confidence intervals included zero; we therefore observed no evidence that cognitive response caution was associated with impulsivity. Average correlations ranged from rho = −0.04 to rho = −0.02 and were consistently low across both subscales and datasets. The 95% confidence intervals for our estimates contain the values of rho that we cannot reject based on our data, the largest (absolute) value of which was observed for the premeditation subscale; rho = −0.04, 95% CI [−0.13, 0.05]. In other words, the largest correlation that our data are consistent with is rho = −0.13, and we can reject the hypothesis of a substantial relationship between response caution and any dimension of self-reported impulsivity. The highest total I^2^ value for any UPPS-P subscale was 30.7%, which is typically interpreted as a low level of heterogeneity (Higgins, Thompson, Deeks, & Altman, 2003) and was not statistically significant (Cochrane's Q = 23.54, *p* = .17). In all cases, this was primarily attributed to variance between datasets rather than within (see Supplementary material B for more details).

## 4 Discussion

Despite conceptual overlap and common terminology, we find no evidence that response caution (or impulsive information processing), as defined by cognitive decision models, is associated with any dimension of impulsivity, as defined by the UPPS-P. It seems that a cautious-impulsive dimension to the way that individuals approach choice reaction time tasks is orthogonal to the propensity for rash action captured by UPPS-P questions.

We extend previous research by attempting to isolate the aspect of cognitive task performance most likely to correlate with self-report impulsivity. Nevertheless, our findings are consistent with several studies and meta-analyses that have shown that self-report measures of impulsivity show weak or no correlation with behavioural tasks (Creswell et al., 2019; Cyders and Coskunpinar, 2011, Cyders and Coskunpinar, 2012; Eisenberg et al., 2019; Saunders, Milyavskaya, Etz, Randles, & Inzlicht, 2018; Schluter, Kim, & Hodgins, 2018; Sharma et al., 2014). Theorists have previously noted the “jingle” problem in impulsivity research (Block, 1995; Sharma, Kohl, Morgan, & Clark, 2013); a common terminology has arisen to describe what may be different underlying constructs. Though it is intuitive to refer to individuals on the low end of the response caution dimension as ‘impulsive’ responders, our results indicate it captures something different from the sub-facets of impulsivity that are captured by the UPPS-P.

The absence of a correlation with self-report does not mean the construct of response caution is not useful. There is evidence that response caution is sensitive to factors such as aging (e.g., Ratcliff et al., 2006; Ratcliff, Thapar, Gomez, & McKoon, 2004), and that it correlates across conflict tasks (Eisenberg et al., 2019;Hedge et al., 2019; Hedge, Powell, et al., 2020). To our knowledge, only one study has examined the relationship between response caution and real-world behaviours associated with self-control (e.g., self-reported drug use, obesity; Eisenberg et al., 2019). They found that factors derived from behavioural tasks, including response caution, showed poor predictive value relative to questionnaire methods (see also Creswell et al., 2019). Other studies have concluded that both behavioural and self-report measures have independent predictive value for real world outcomes (Sharma et al., 2014). Future efforts to resolve these inconsistencies may benefit from the dissociating response caution from other processes of potential interest in commonly used tasks, such as conflict control and processing speed (Hedge, Powell, et al., 2020). However, while we may gain a better understanding of these tasks through the application of cognitive models, this will not automatically lead us to better real-world prediction (for a discussion, see Hedge, Bompas, & Sumner, 2020).

Though our analysis was not exhaustive given the range of impulsivity measures used in the literature (Sharma et al., 2014), we expect our findings are not specific to the UPPS-P. The UPPS-P was designed to capture factors identified in existing impulsivity questionnaires (Whiteside & Lynam, 2001), and meta-analyses have shown that the UPPS-P and other questionnaires reflect common underlying traits (Sharma et al., 2014). With regard to behavioural tasks, as we have noted, response caution parameters correlate across conflict tasks, both in our data and in a larger battery of tasks (Eisenberg et al., 2019; Hedge, Powell, et al., 2020). We therefore have no reason to expect that response caution would correlate with self-report had we used different tasks, at least when considering spontaneous strategies in a healthy population. Cyders and Coskunpinar (2012) observed small but significant correlations with self-report measures and behavioural measures taken from “prepotent response inhibition” tasks (including the stop-signal, antisaccade, and continuous performance tasks), though variance in these measures may also reflect multiple underlying sources. For example, a recent model-based analysis of the stop-signal task suggests that correlations with self-reported impulsivity may reflect attentional lapses rather than inhibition (Skippen et al., 2019).

If people's disposition to make decisions quickly at the expense of errors is not driven by impulsivity, as commonly understood, then what does it reflect? It is possible that response caution is a behavioural “snap shot” (Cyders & Coskunpinar, 2012), and captures something different to individuals' self-perceived caution in everyday life. However, it appears that individuals are consistent in how they strategically approach similar tasks, given the between-task and reliability correlations we have previously observed (Hedge et al., 2019; Hedge, Powell, et al., 2020). We assume that individuals have some control over their level of caution - if we instruct participants to respond more quickly, they are able to do so, and this is captured in part by lowering their boundary (though other parameters can also change; Rae, Heathcote, Donkin, Averell, & Brown, 2014). We instructed participants to be both fast and accurate in our studies, except for the additional speed- and accuracy-emphasis blocks in Datasets 4 and 5, and we asked whether their default strategy might reflect trait impulsivity. There is evidence that individuals typically favour accuracy over speed by default (Forstmann et al., 2008), though we know little about why they spontaneously adopt the levels of caution that they do. Notably, while evidence accumulation models mathematically dissociate caution from parameters that represent processing ability, they may be correlated in real data (Schmiedek et al., 2007). Individuals who have higher levels of ability (higher drift rates) can achieve the same level of accuracy as individuals with lower ability (lower drift rates) while setting a lower boundary. Advancing our understanding of the mechanisms underlying response caution may benefit from examining why individuals adopt sub-optimal strategies for their level of ability, or by incentivising participants to favour speed or accuracy (e.g., to optimise a reward; Starns & Ratcliff, 2010), rather than looking at what they do spontaneously.

In conclusion, we show that individuals with a tendency to respond quickly while risking errors do not self-report higher levels of impulsivity. These findings inform the interpretation of individual differences in response caution in domains such as aging (Ratcliff et al., 2006) and neuropsychological conditions (Metin et al., 2013; Powell et al., 2019). They are also a further illustration that the relationship between lab-based and self-report measures of impulsivity is not straightforward (Cyders & Coskunpinar, 2012; Sharma et al., 2014; Wennerhold & Friese, 2020).

## CRediT authorship contribution statement

**Craig Hedge:**Conceptualization, Methodology, Formal analysis, Writing - original draft, Visualization, Investigation.**Georgina Powell:**Conceptualization, Writing - review & editing.**Aline Bompas:**Conceptualization, Writing - review & editing.**Petroc Sumner:**Conceptualization, Writing - review & editing, Funding acquisition, Supervision.

## Appendix A Supplementary data

Supplementary material Image 1
