# Supplementary material for: Self-reported impulsivity does not predict response caution
Source: Pers Individ Dif. 2020 Dec 1;167:110257. doi: 10.1016/j.paid.2020.110257 (PMC7457714; doi:10.1016/j.paid.2020.110257)
Supplement: Supplementary file 1 — Supplementary material [file mmc1.docx]

**Supplementary Material A: Description of drift diffusion model and diffusion model for conflict tasks**

In this section, we describe the models that we applied in our analysis in more detail. **Drift diffusion model**

The drift diffusion model (Ratcliff, 1978) assumes that the decision phase can be represented by the noisy accumulation of evidence to a boundary (see Figure A1). The model has three main parameters- drift rate (v), boundary separation (a) and non-decision time (Ter). The drift rate parameter represents the quality of information extracted from the stimulus, which can depend on both the stimulus (e.g. higher coherence in a dot motion task) and the individual (e.g. higher processing efficiency). Boundary separation or ‘response caution’ reflects the amount of evidence required before making a response. A period of perceptual encoding and motor execution are assumed to precede and follow the decision phase respectively, which are collectively referred to as non-decision time. When trial types are randomly intermixed in a design, for example low- and high-coherence trials in a dot motion task, it is commonly assumed that boundary separation and non-decision time do not vary between conditions.

In addition to the main parameters, the model can also include a response bias parameter (z). A task may elicit a response bias if one response option is more common than another (e.g. if more trials are administered in which the direction of coherent motion is left). Further, parameters can be included that capture intertrial variability in drift rates (eta), the start point of the evidence accumulation process (sZ), and non-decision time (sTer). In our fits, we assumed that drift rates were normally distributed across trials, and sZ and sTer were uniformly distributed. We assumed that the decision process was unbiased (z=a/2).

For the fits to the random dot motion task (Dataset 5) reported in text, we estimated eight parameters for each participant (see Hedge et al., 2019 for full details):

1. Boundary separation under speed emphasis
2. Boundary separation under standard instructions
3. Boundary separation under accuracy emphasis
4. Drift rate for high coherence trials
5. Drift rate for low coherence trials
6. Mean non-decision time
7. Starting point variability
8. Non-decision variability

Note that unless indicated, the parameters are constrained to be equal across trial type (high coherence, low coherence) or instruction condition (speed, standard or accuracy emphasis).


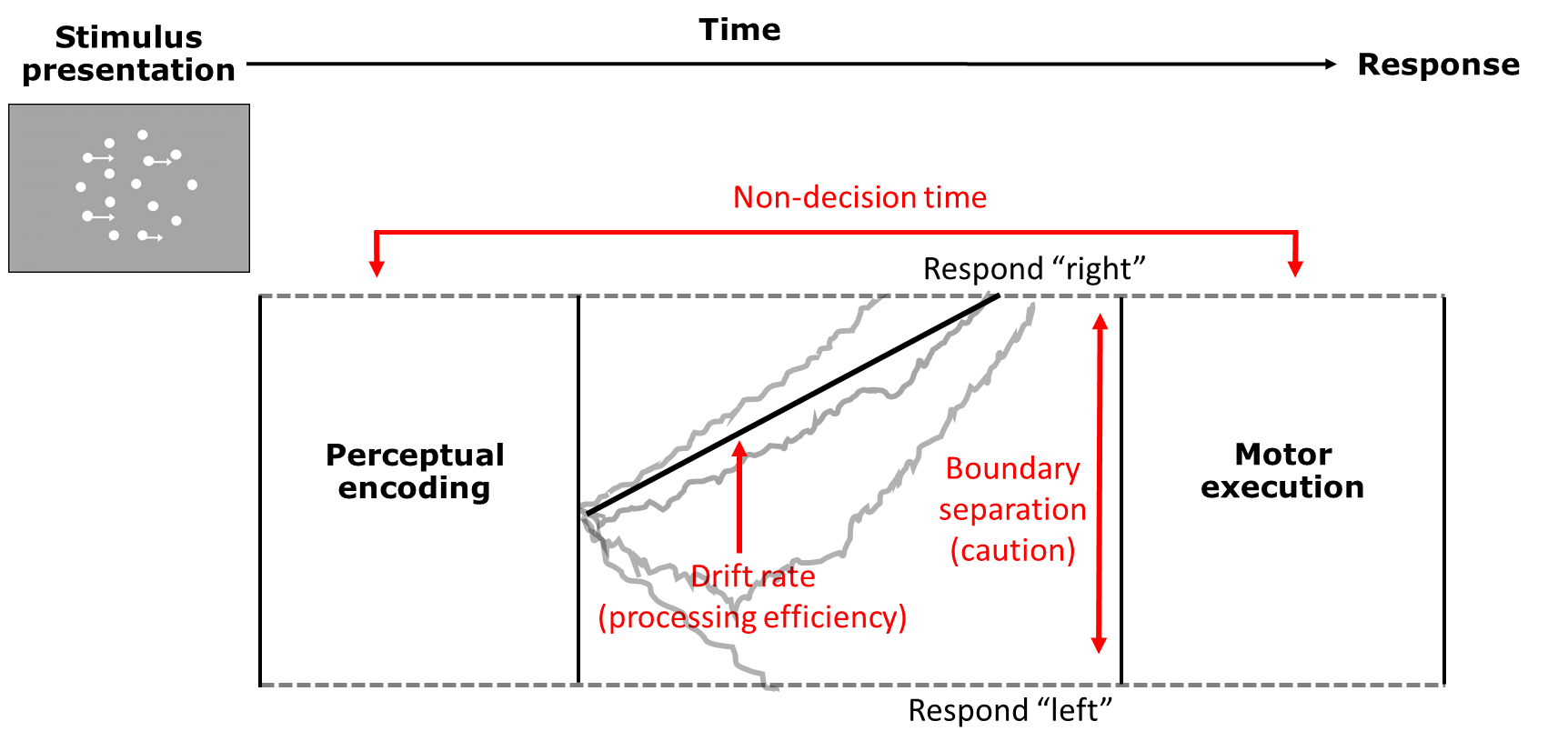


**Figure A1. Schematic of the drift-diffusion model. See text for details.**

**Diffusion model for conflict tasks**

The diffusion model for conflict tasks DMC; Ulrich et al., 2015) is an extension of the drift-diffusion model, designed to account for patterns of data observed in conflict tasks. It assumes that automatic activation (e.g. reading the word in the Stroop task) and deliberate processing (e.g. naming the font colour in the Stroop task) are processed through separate routes. The overall evidence accumulation is a composite of activation in these two routes (see Figure A2).

The DMC shares several parameters with the standard drift-diffusion model (boundary separation, non-decision time, intertrial variability). The drift rate parameter in the DMC represents the efficiency of controlled information processing. This corresponds to the processing of (e.g.) the font colour in the Stroop task, and it is assumed to be constant across congruent, neutral and incongruent conditions. The difference between congruency conditions is captured by the presence of automatic activation, which facilitates the correct response in congruent trials and has the opposite effect in incongruent trials. Automatic activation is implemented as a rescaled gamma function defined by amplitude (the height it reaches), shape and scale parameters. In the original implementation and our own, the shape parameter is fixed to a constant (2) and the scale parameter is referred to as the time-to-peak of the automatic activation.


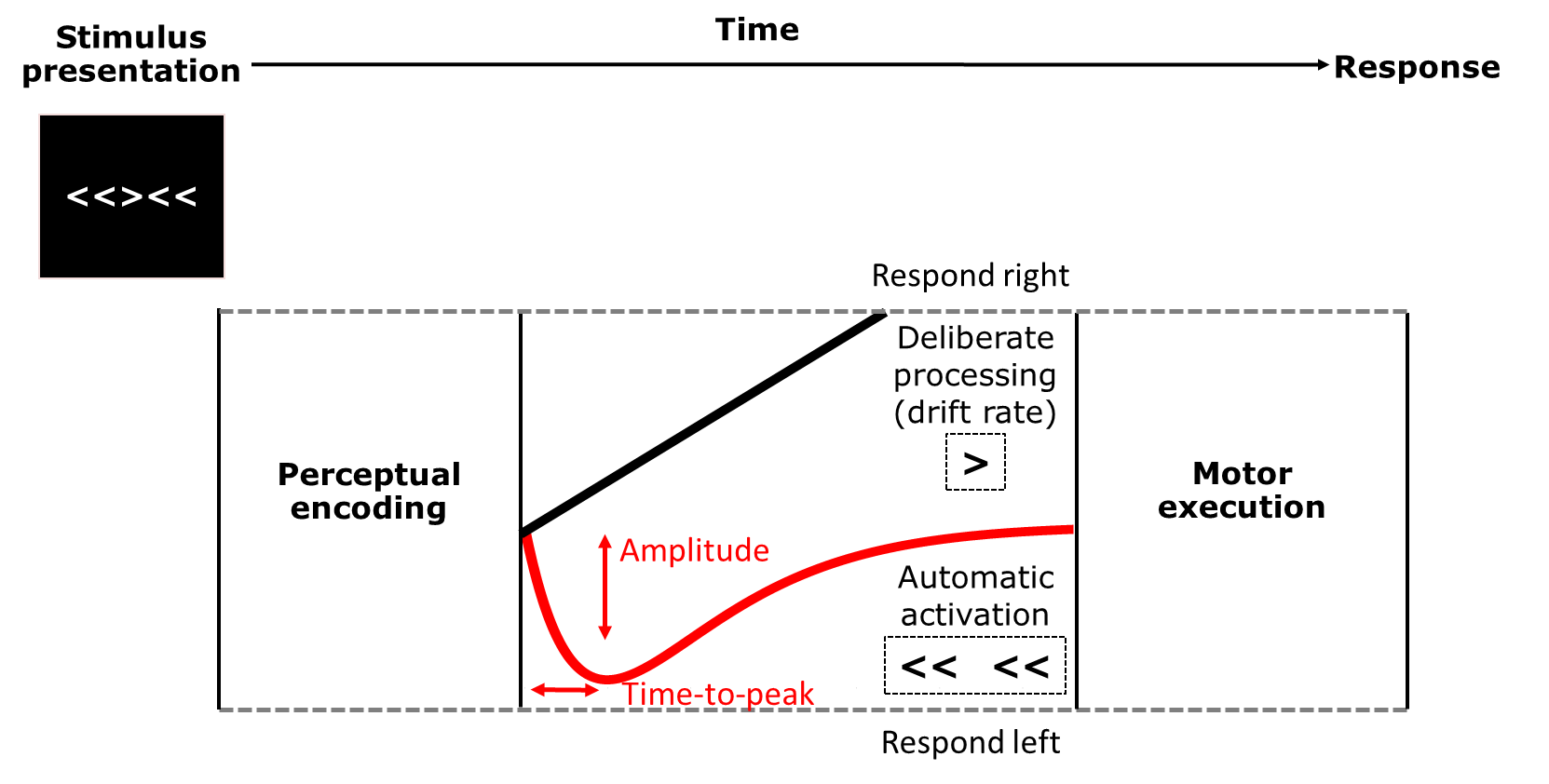


**Figure A2. Schematic of the diffusion model for conflict tasks. See text for details.**

Our DMC modelling thus consisted of 7 basic parameters

1. Boundary separation
2. Drift rate
3. Amplitude of automatic activation (-Amplitude for incongruent trials, 0 for neutral trials where administered)
4. Time to peak of automatic activation
5. Mean non-decision time (normal distribution)
6. Starting point variability (beta distribution)
7. Non-decision variability (normal distribution)

For the version of the Simon task in Dataset 3 in which congruent and incongruent trials were administered in separate blocks, we estimated separate boundary separation values each condition. In the speed-accuracy trade-off experiments (Datasets 4 and 5), we estimated separate boundary separation values for speed-, standard-, and accuracy-emphasis trials.

**Supplementary Material B: Individual correlations and other parameters**

In this section, we report the individual correlations that are included in our meta-analysis in the main text and plotted in Figure 3 (Table B1).We also perform the same analysis on other key parameters of the model. Note that the amplitude and time-to-peak of automatic activation parameters are unique to the diffusion model for conflict tasks, so we do not report correlations for those parameters in the random dot motion task in Dataset 5. See Supplementary Material A for a description of each parameter.

**Table B1. Correlations between boundary separation and UPPS-P subscales**

| Dataset | Task | N | NegU | PreMed | Pers | SenSeek | PosU |
| --- | --- | --- | --- | --- | --- | --- | --- |
| 1 | Flanker | 50 | 0.07 | -0.08 | 0.01 | -0.12 | 0.02 |
| 1 | Simon | 50 | 0.14 | -0.06 | -0.02 | -0.12 | 0 |
| 2 | Flanker | 103 | 0.03 | 0.03 | 0.13 | -0.01 | -0.06 |
| 2 | Stroop | 103 | -0.07 | -0.02 | 0.04 | -0.04 | -0.02 |
| 3 | Simon_Intermixed | 102 | -0.09 | -0.05 | -0.05 | -0.05 | -0.1 |
| 3 | Simon_Congruent | 102 | 0 | -0.05 | -0.06 | -0.11 | -0.05 |
| 3 | Simon_Incongruent | 102 | 0.02 | 0.06 | -0.07 | 0.05 | 0.04 |
| 4 | Flanker_Speed | 43 | -0.26 | -0.15 | **-0.38*** | 0.03 | -0.09 |
| 4 | Flanker_Standard | 43 | -0.14 | -0.15 | -0.3 | 0.12 | -0.17 |
| 4 | Flanker_Accuracy | 43 | -0.06 | -0.14 | -0.03 | 0.19 | -0.1 |
| 4 | Stroop_Speed | 43 | -0.02 | -0.22 | -0.2 | -0.12 | 0.1 |
| 4 | Stroop_Standard | 43 | -0.12 | -0.17 | -0.03 | -0.05 | -0.1 |
| 4 | Stroop_Accuracy | 43 | -0.18 | -0.27 | 0.06 | -0.14 | -0.24 |
| 5 | Flanker_Speed | 69 | 0.12 | 0.06 | **0.23*** | -0.13 | 0.11 |
| 5 | Flanker_Standard | 69 | -0.07 | -0.05 | -0.05 | 0.03 | -0.04 |
| 5 | Flanker_Accuracy | 69 | -0.07 | 0.1 | -0.07 | 0.12 | -0.09 |
| 5 | RDM_Speed | 69 | 0.01 | 0.12 | 0.16 | -0.21 | 0.15 |
| 5 | RDM_Standard | 69 | -0.19 | 0.08 | 0.01 | -0.15 | -0.11 |
| 5 | RDM_Accuracy | 69 | -0.03 | 0 | -0.03 | 0 | 0.01 |

*p<05

**Table B2. Summary of meta-analysis of correlations between boundary separation/response caution and the UPPS-P impulsivity questionnaire subscales.**

| Subscale | rho | Lower 95% CI | Upper 95 % CI | p | I2 between datasets (%) | I2 within datasets (%) | Cochrane's Q | p |
| --- | --- | --- | --- | --- | --- | --- | --- | --- |
| Positive Urgency | -0.03 | -0.09 | 0.02 | 0.22 | 0 | 0 | 9.99 | 0.93 |
| Sensation seeking | -0.04 | -0.09 | 0.02 | 0.16 | 0 | 0 | 12.32 | 0.83 |
| (lack of) Perseverance | -0.02 | -0.11 | 0.07 | 0.65 | 30.67 | 0 | 23.54 | 0.17 |
| (lack of) Premeditation | -0.04 | -0.13 | 0.05 | 0.41 | 28.55 | 0 | 12.92 | 0.80 |
| Negative Urgency | -0.04 | -0.09 | 0.01 | 0.15 | 0 | 0 | 11.53 | 0.87 |

**Table B3. Correlations between amplitude and UPPS-P subscales**

| Dataset | Task | N | NegU | PreMed | Pers | SenSeek | PosU |
| --- | --- | --- | --- | --- | --- | --- | --- |
| 1 | Flanker | 50 | -0.06 | 0.26 | 0.15 | 0.22 | 0.12 |
| 1 | Simon | 50 | -0.11 | -0.08 | **-0.39**** | -0.15 | **-0.28*** |
| 2 | Flanker | 103 | -0.05 | -0.04 | -0.01 | 0.02 | -0.03 |
| 2 | Stroop | 103 | 0.08 | -0.06 | -0.17 | 0.12 | 0.01 |
| 3 | Simon_Intermixed | 102 | -0.01 | -0.01 | -0.07 | -0.04 | 0.03 |
| 3 | Simon_Congruent | 102 | -0.04 | 0.05 | 0.06 | 0.08 | -0.1 |
| 4 | Flanker_SAT | 43 | 0.19 | -0.2 | 0.12 | -0.01 | 0.17 |
| 4 | Stroop_SAT | 43 | -0.06 | 0.08 | -0.21 | 0.18 | -0.13 |
| 5 | Flanker_SAT | 69 | -0.15 | -0.03 | 0.05 | -0.14 | -0.1 |

**p<.01, *p<.05

**Table B4. Summary of meta-analysis of correlations between amplitude and the UPPS-P impulsivity questionnaire subscales.**

| Subscale | rho | Lower 95% CI | Upper 95 % CI | p | I2 between datasets (%) | I2 within datasets (%) | Cochrane's Q | p |
| --- | --- | --- | --- | --- | --- | --- | --- | --- |
| Positive Urgency | -0.04 | -0.11 | 0.04 | 0.34 | 0 | 0 | 8.19 | 0.42 |
| Sensation seeking | 0.03 | -0.04 | 0.11 | 0.38 | 0 | 0 | 8.53 | 0.38 |
| (lack of) Perseverance | -0.06 | -0.16 | 0.05 | 0.3 | 0 | 50.07 | 15.96 | 0.04 |
| (lack of) Premeditation | -0.01 | -0.08 | 0.07 | 0.89 | 0 | 0 | 6.79 | 0.56 |
| Negative Urgency | -0.02 | -0.1 | 0.05 | 0.58 | 0 | 0 | 4.87 | 0.77 |

**
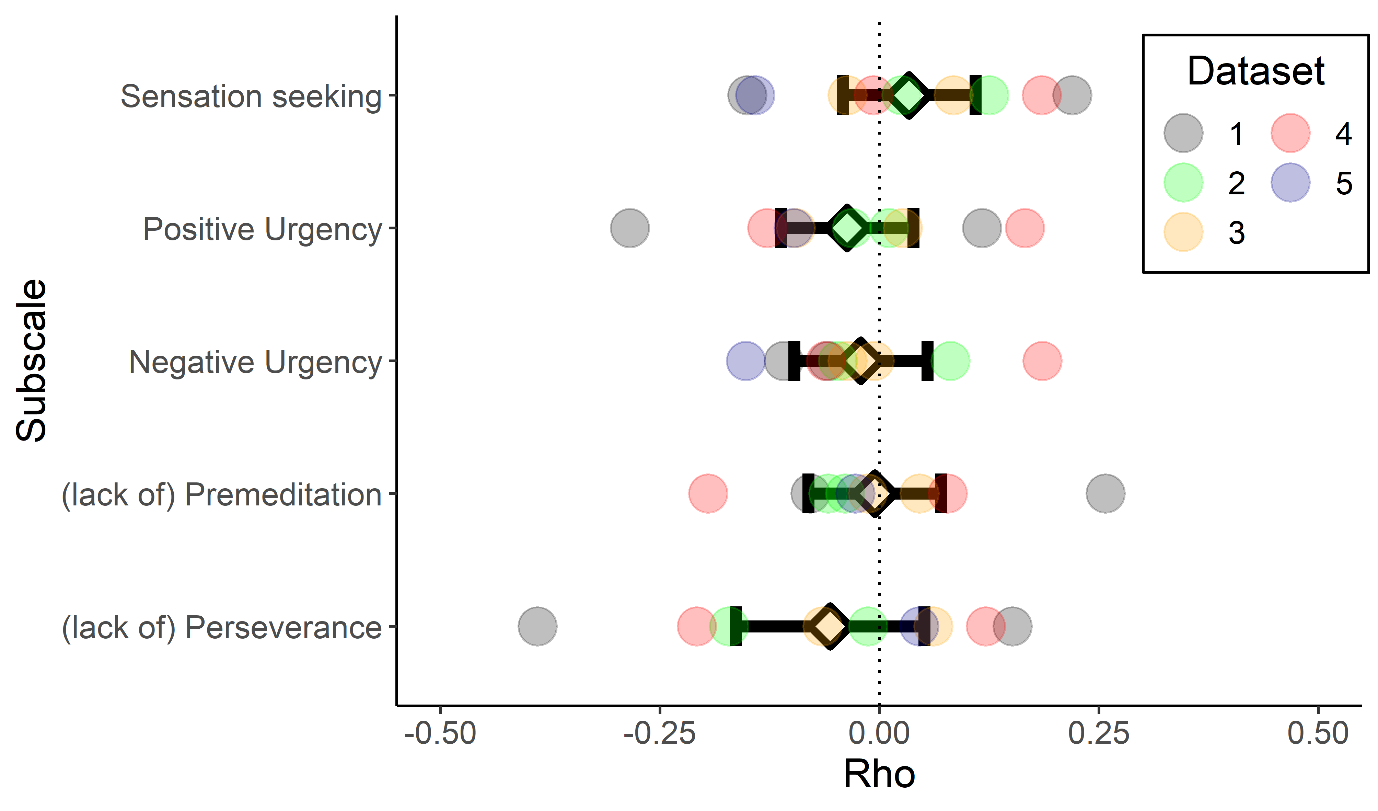
**

**Figure B1.** *Meta-analytic (black diamonds) and observed (circles) correlations between the amplitude parameter and the UPPS-P impulsivity questionnaire subscales. Error bars show 95% confidence intervals. A multi-level random effects meta-analysis was performed on Spearman’s rho correlations calculated for each pair of tasks, allowing for clustering where multiple correlations were taken from the same dataset. Note that all the 95% confidence intervals include zero.*

**Table B5. Correlations between Time to peak and UPPS-P subscales**

| Dataset | Task | N | NegU | PreMed | Pers | SenSeek | PosU |
| --- | --- | --- | --- | --- | --- | --- | --- |
| 1 | Flanker | 50 | -0.18 | -0.01 | -0.08 | 0.1 | -0.21 |
| 1 | Simon | 50 | 0.03 | -0.07 | -0.15 | -0.13 | -0.01 |
| 2 | Flanker | 103 | 0 | -0.04 | -0.02 | 0.07 | -0.02 |
| 2 | Stroop | 103 | 0.07 | 0.14 | 0.04 | 0.01 | 0.02 |
| 3 | Simon_Intermixed | 102 | 0.01 | 0.07 | -0.03 | 0.02 | -0.08 |
| 3 | Simon_Congruent | 102 | -0.06 | -0.02 | -0.04 | 0.04 | -0.18 |
| 4 | Flanker_SAT | 43 | 0.12 | 0 | 0.15 | -0.1 | 0.12 |
| 4 | Stroop_SAT | 43 | -0.05 | -0.01 | -0.02 | 0.13 | -0.2 |
| 5 | Flanker_SAT | 69 | -0.01 | **0.29*** | 0.14 | 0.17 | 0.08 |

*p<.05

**Table B6. Summary of meta-analysis of correlations between Time to peak and the UPPS-P impulsivity questionnaire subscales.**

| Subscale | rho | Lower 95% CI | Upper 95 % CI | p | I2 between datasets (%) | I2 within datasets (%) | Cochrane's Q | p |
| --- | --- | --- | --- | --- | --- | --- | --- | --- |
| Positive Urgency | -0.05 | -0.13 | 0.02 | 0.16 | 0 | 0 | 7.23 | 0.51 |
| Sensation seeking | 0.04 | -0.04 | 0.11 | 0.33 | 0 | 0 | 4.19 | 0.84 |
| (lack of) Perseverance | 0 | -0.08 | 0.07 | 0.93 | 0 | 0 | 4.34 | 0.83 |
| (lack of) Premeditation | 0.05 | -0.03 | 0.13 | 0.2 | 0 | 5.84 | 7.71 | 0.46 |
| Negative Urgency | -0.01 | -0.08 | 0.07 | 0.87 | 0 | 0 | 3.33 | 0.91 |

**
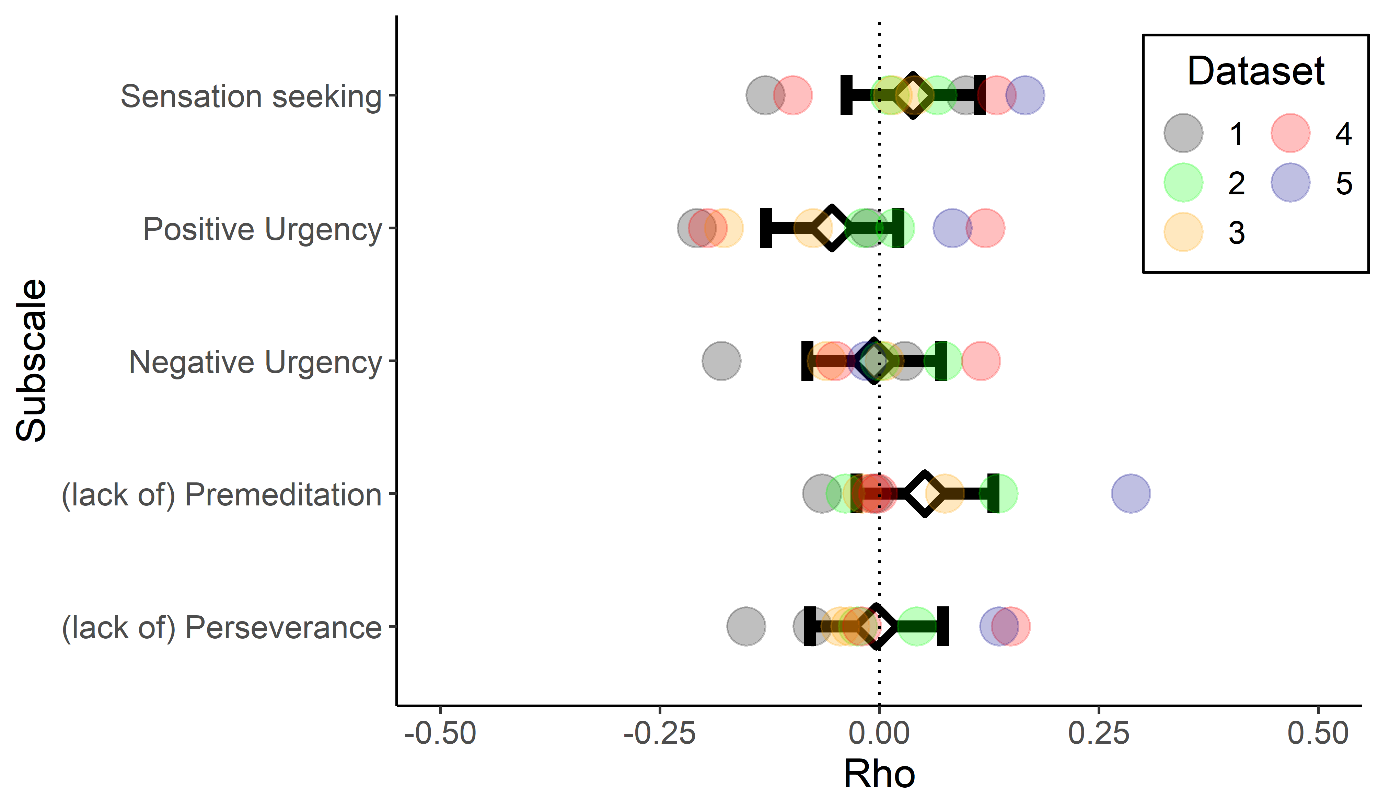
**

**Figure B2.** *Meta-analytic (black diamonds) and observed (circles) correlations between the time-to-peak parameter and the UPPS-P impulsivity questionnaire subscales. Error bars show 95% confidence intervals. A multi-level random effects meta-analysis was performed on Spearman’s rho correlations calculated for each pair of tasks, allowing for clustering where multiple correlations were taken from the same dataset. Note that all the 95% confidence intervals include zero.*

**Table B7. Correlations between drift rate and UPPS-P subscales**

| Dataset | Task | N | NegU | PreMed | Pers | SenSeek | PosU |
| --- | --- | --- | --- | --- | --- | --- | --- |
| 1 | Flanker | 50 | 0 | 0.15 | **0.28*** | 0.09 | 0.05 |
| 1 | Simon | 50 | -0.16 | -0.13 | 0.01 | 0.1 | -0.07 |
| 2 | Flanker | 103 | -0.1 | -0.19 | **-0.27**** | -0.11 | 0 |
| 2 | Stroop | 103 | 0.09 | -0.02 | 0 | -0.01 | 0.14 |
| 3 | Simon_Intermixed | 102 | 0.11 | 0.05 | 0.07 | 0.01 | 0.17 |
| 3 | Simon_Congruent | 102 | -0.08 | -0.02 | -0.02 | -0.03 | -0.03 |
| 4 | Flanker_SAT | 43 | 0.06 | -0.14 | 0.21 | -0.05 | 0.19 |
| 4 | Stroop_SAT | 43 | 0.06 | -0.09 | 0.12 | 0.07 | -0.19 |
| 5 | Flanker_SAT | 69 | -0.07 | -0.17 | -0.2 | -0.04 | -0.13 |
| 5 | RDM_Congruent | 69 | 0.02 | -0.09 | 0.06 | -0.06 | -0.05 |
| 5 | RDM_Incongruent | 69 | -0.05 | -0.12 | 0.1 | -0.01 | -0.14 |

**p<.01, *p<.05

**Table B8. Summary of meta-analysis of correlations between drift rate and the UPPS-P impulsivity questionnaire subscales.**

| Subscale | rho | Lower 95% CI | Upper 95 % CI | p | I2 between datasets (%) | I2 within datasets (%) | Cochrane's Q | p |
| --- | --- | --- | --- | --- | --- | --- | --- | --- |
| Positive Urgency | 0.01 | -0.07 | 0.09 | 0.88 | 9.38 | 7.13 | 11.50 | 0.32 |
| Sensation seeking | -0.01 | -0.08 | 0.05 | 0.68 | 0 | 0 | 2.72 | 0.99 |
| (lack of) Perseverance | 0.02 | -0.09 | 0.12 | 0.73 | 9.35 | 43.03 | 20.70 | 0.02 |
| (lack of) Premeditation | -0.07 | -0.14 | 0 | 0.06 | 0 | 0 | 7.62 | 0.67 |
| Negative Urgency | -0.01 | -0.08 | 0.06 | 0.79 | 0 | 0 | 5.82 | 0.83 |

**
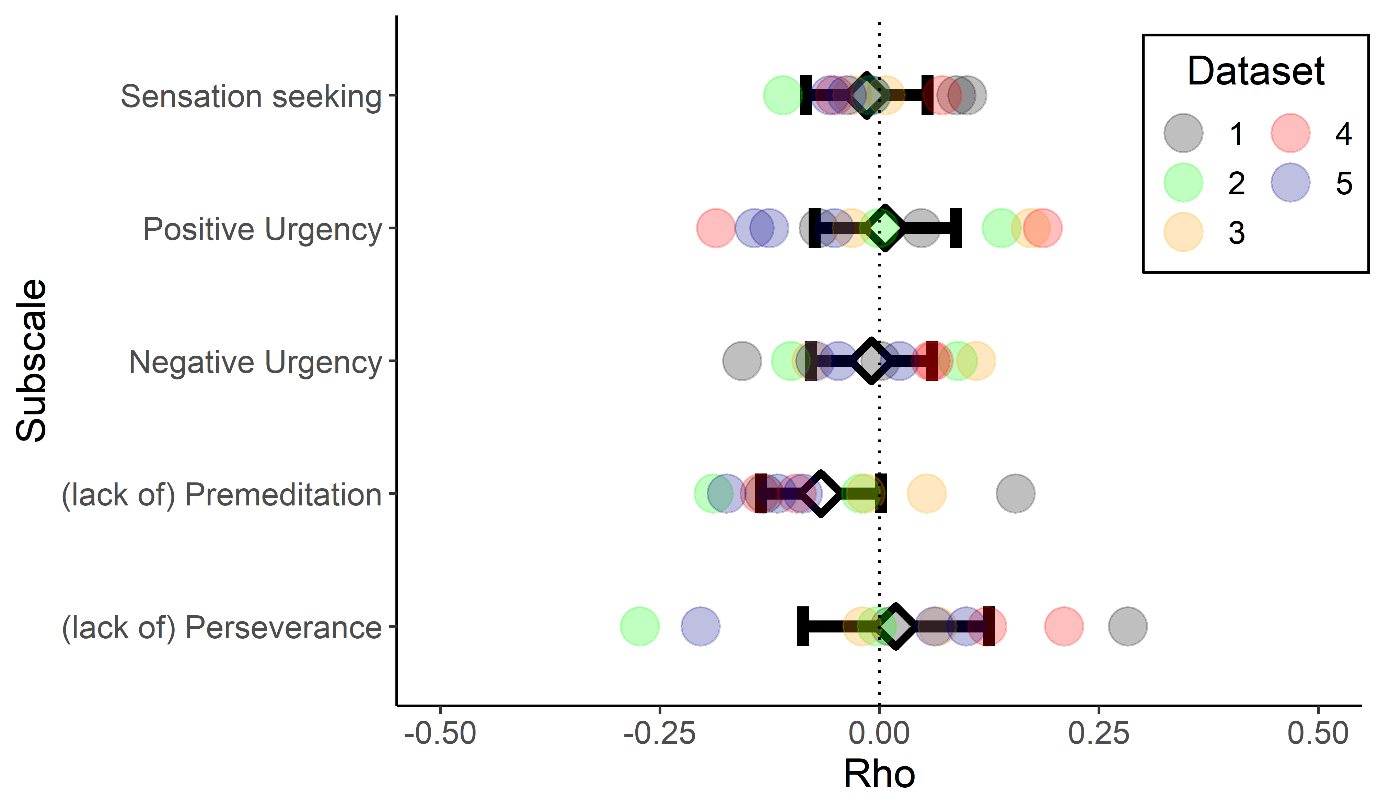
**

**Figure B3.** *Meta-analytic (black diamonds) and observed (circles) correlations between the drift rate parameter and the UPPS-P impulsivity questionnaire subscales. Error bars show 95% confidence intervals. A multi-level random effects meta-analysis was performed on Spearman’s rho correlations calculated for each pair of tasks, allowing for clustering where multiple correlations were taken from the same dataset. Note that all the 95% confidence intervals include zero.*

**Table B9. Correlations between non-decision time and UPPS-P subscales**

| Dataset | Task | N | NegU | PreMed | Pers | SenSeek | PosU |
| --- | --- | --- | --- | --- | --- | --- | --- |
| 1 | Flanker | 50 | 0.19 | 0.06 | 0.05 | -0.1 | 0.05 |
| 1 | Simon | 50 | 0.17 | -0.06 | 0.2 | -0.1 | 0.02 |
| 2 | Flanker | 103 | -0.02 | 0.02 | 0.14 | -0.06 | 0 |
| 2 | Stroop | 103 | 0.02 | 0.05 | 0.15 | -0.02 | 0.05 |
| 3 | Simon_Intermixed | 102 | 0.02 | 0.17 | -0.08 | 0.17 | 0.07 |
| 3 | Simon_Congruent | 102 | 0.01 | 0.14 | 0.02 | 0.05 | 0.06 |
| 4 | Flanker_SAT | 43 | -0.11 | -0.04 | 0.17 | **0.30*** | -0.01 |
| 4 | Stroop_SAT | 43 | -0.13 | 0.01 | -0.11 | 0.24 | -0.03 |
| 5 | Flanker_SAT | 69 | -0.04 | -0.08 | 0.05 | 0.04 | -0.1 |
| 5 | RDM_SAT | 69 | 0.16 | -0.04 | 0.11 | -0.04 | 0.02 |

*p<.05

**Table B10. Summary of meta-analysis of correlations between non-decision time and the UPPS-P impulsivity questionnaire subscales.**

| Subscale | rho | Lower 95% CI | Upper 95 % CI | p | I2 between datasets (%) | I2 within datasets (%) | Cochrane's Q | p |
| --- | --- | --- | --- | --- | --- | --- | --- | --- |
| Positive Urgency | 0.02 | -0.05 | 0.09 | 0.57 | 0 | 0 | 1.73 | 1.00 |
| Sensation seeking | 0.05 | -0.07 | 0.16 | 0.45 | 44.51 | 0 | 11.09 | 0.27 |
| (lack of) Perseverance | 0.07 | -0.01 | 0.15 | 0.07 | 7.15 | 0 | 6.58 | 0.68 |
| (lack of) Premeditation | 0.03 | -0.05 | 0.12 | 0.41 | 13.55 | 0 | 5.21 | 0.82 |
| Negative Urgency | 0.03 | -0.04 | 0.1 | 0.45 | 0 | 0 | 6.05 | 0.73 |

**
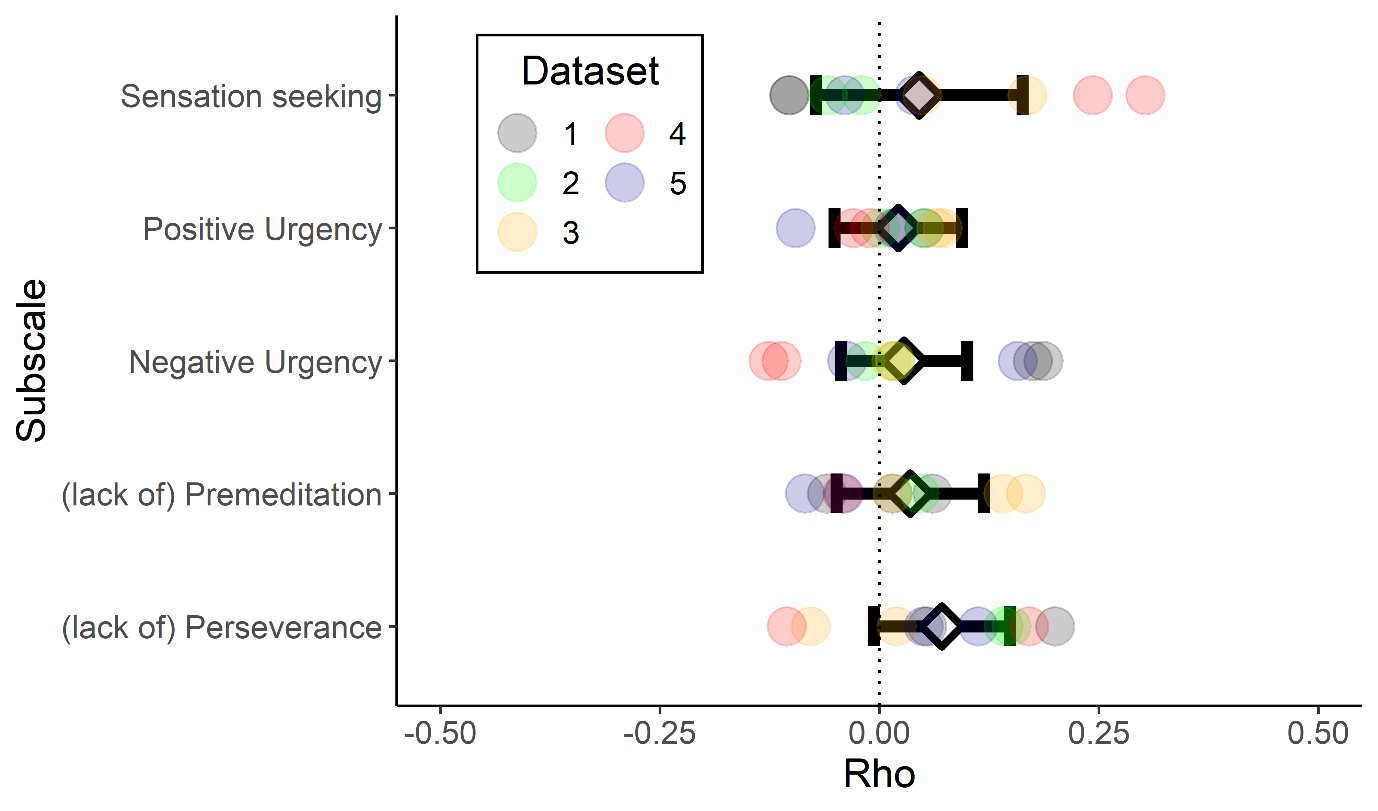
**

**Figure B4.** *Meta-analytic (black diamonds) and observed (circles) correlations between the non-decision parameter and the UPPS-P impulsivity questionnaire subscales. Error bars show 95% confidence intervals. A multi-level random effects meta-analysis was performed on Spearman’s rho correlations calculated for each pair of tasks, allowing for clustering where multiple correlations were taken from the same dataset. Note that all the 95% confidence intervals include zero.*
